# Application of a three-dimensional printed pelvic model in laparoscopic radical resection of rectal cancer

**DOI:** 10.3389/fonc.2023.1195404

**Published:** 2023-06-19

**Authors:** Feng Lu, Lei Qiu, Peng Yu, Da-Lai Xu, Yong-Chang Miao, Gang Wang

**Affiliations:** Department of General Surgery, Second People’s Hospital of Lianyungang, Lianyungang, Jiangsu, China

**Keywords:** rectal cancer, three-dimensional printer, pelvic, inferior mesenteric artery (IMA), laparoscopic surgery

## Abstract

**Introduction:**

To investigate the application value of a three-dimensional (3D) printed pelvic model in laparoscopic radical resection of rectal cancer.

**Methods:**

Clinical data of patients undergoing laparoscopic radical rectal cancer surgery in The Second People's Hospital of Lianyungang City from May 2020 to April 2022 were selected. Patients were randomly divided into general imaging examination group (control group, n=25) and 3D printing group (observation group, n=25) by random number table method, and the perioperative situation of patients in the two groups was compared.

**Results:**

There was no significant difference in general data between the two groups (p>0.05). Operation time, intraoperative blood loss, intraoperative time to locate inferior mesenteric artery, intraoperative time to locate left colic artery, first postoperative exhaust time and length of hospital stay in the observation group were all lower than those in the control group (P < 0.05); There were no significant differences in the total number of lymph nodes and complications between the two groups (P > 0.05).

**Discussion:**

The application of 3D printed pelvic model in laparoscopic radical resection of rectal cancer is conducive to understanding pelvic structure and mesenteric vascular anatomy, reducing intraoperative bleeding and shortening operation time, which is worthy of further clinical application.

## Introduction

Colorectal cancer is the second leading cause of cancer-related mortality worldwide (1). Rectal cancer constitutes approximately 30% of all colorectal cancers. Comprehensive treatment based on surgery can improve the prognosis of patients (2, 3). High ligation refers to the vascular root ligation before the inferior mesenteric artery sends off the left colic artery, and low ligation refers to the ligation after the inferior mesenteric artery sends off the left colic artery, i.e. the left colic artery is preserved. For a long time, there has been no conclusion on how to select the ligation method, but a large number of studies (4–6) have shown that low ligation can improve anastomotic perfusion, reduce the incidence of anastomotic leak, and improve the prognosis of patients. Therefore, it is particularly important to conduct accurate dissection of inferior mesenteric artery and its branches in limited visual field during laparoscopic radical resection of rectal cancer. However, the anatomy of abdominal surgery is complex and the degree of variation is high. 3D printing technology can be used for preoperative simulation and intraoperative navigation, so as to help doctors choose the appropriate surgical plan. Currently, it has been widely used in the fields of hepatobiliary surgery (7), pancreatic surgery (8), orthopedics (9) and urology surgery (10, 11), but it is still in its infancy in the field of rectal cancer surgery. This study studied the clinical data of 50 patients undergoing laparoscopic radical resection of rectal cancer treated in our department from May 2020 to April 2022, and discussed the application value of 3D printing pelvic model in laparoscopic radical resection of rectal cancer, so as to provide clinical reference.

## Materials and methods

### Study design and participants

Clinical data of 50 patients undergoing laparoscopic radical resection for rectal cancer were selected and randomly divided into general imaging examination group (control group: 25 cases) and 3D printing group (observation group: 25 cases) by random number table method. All patients underwent abdominal and pelvic plain scan and double-phase enhanced scan before surgery. The observation group imported CT image data into 3D modeling software in DICOM format for 3D printing. According to the results of imaging examination and 3D printing model, preoperative evaluation and operation plan were made. This study was approved by the Ethics Committee of our hospital, and all patients and their families signed informed consent before surgery.

### Inclusion criteria and exclusion criteria

Inclusion criteria: (1) Preoperative pathological diagnosis of rectal cancer. (2) Laparoscopic surgery, no distant metastasis was found, resection margin was negative (R0), and postoperative pathological data was complete. Exclusion criteria: (1) Due to abdominal adhesion and intraoperative bleeding, the vascular anatomy could not be judged. (2) Emergency surgery due to obstruction or bleeding. (3) Complicated with abnormal functions of heart, liver and kidney. (4) Incomplete case data.

### Three dimensional pelvic model

**Figure 1:**
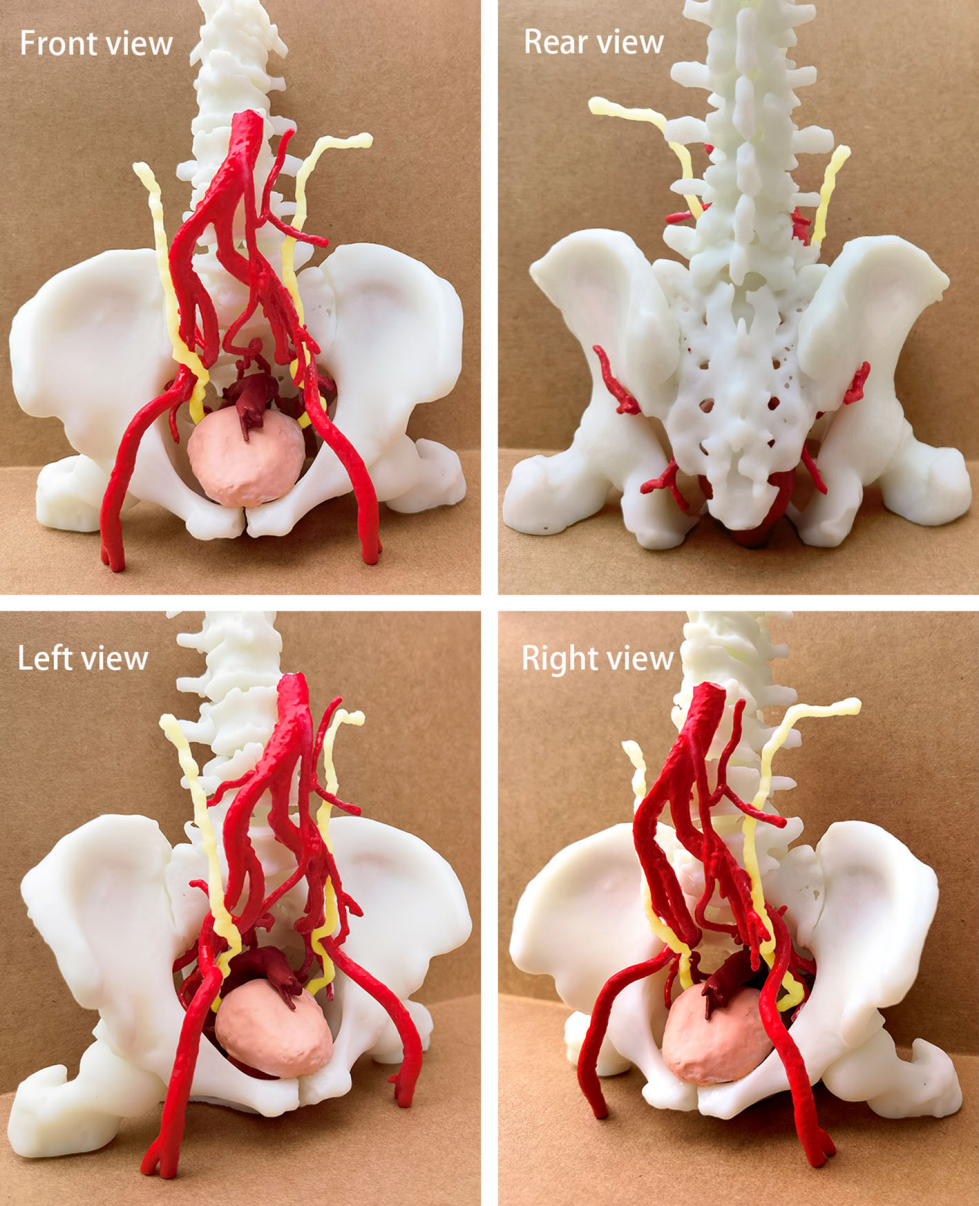
Quadrilateral view of the pelvic model.

**Figure 2:**
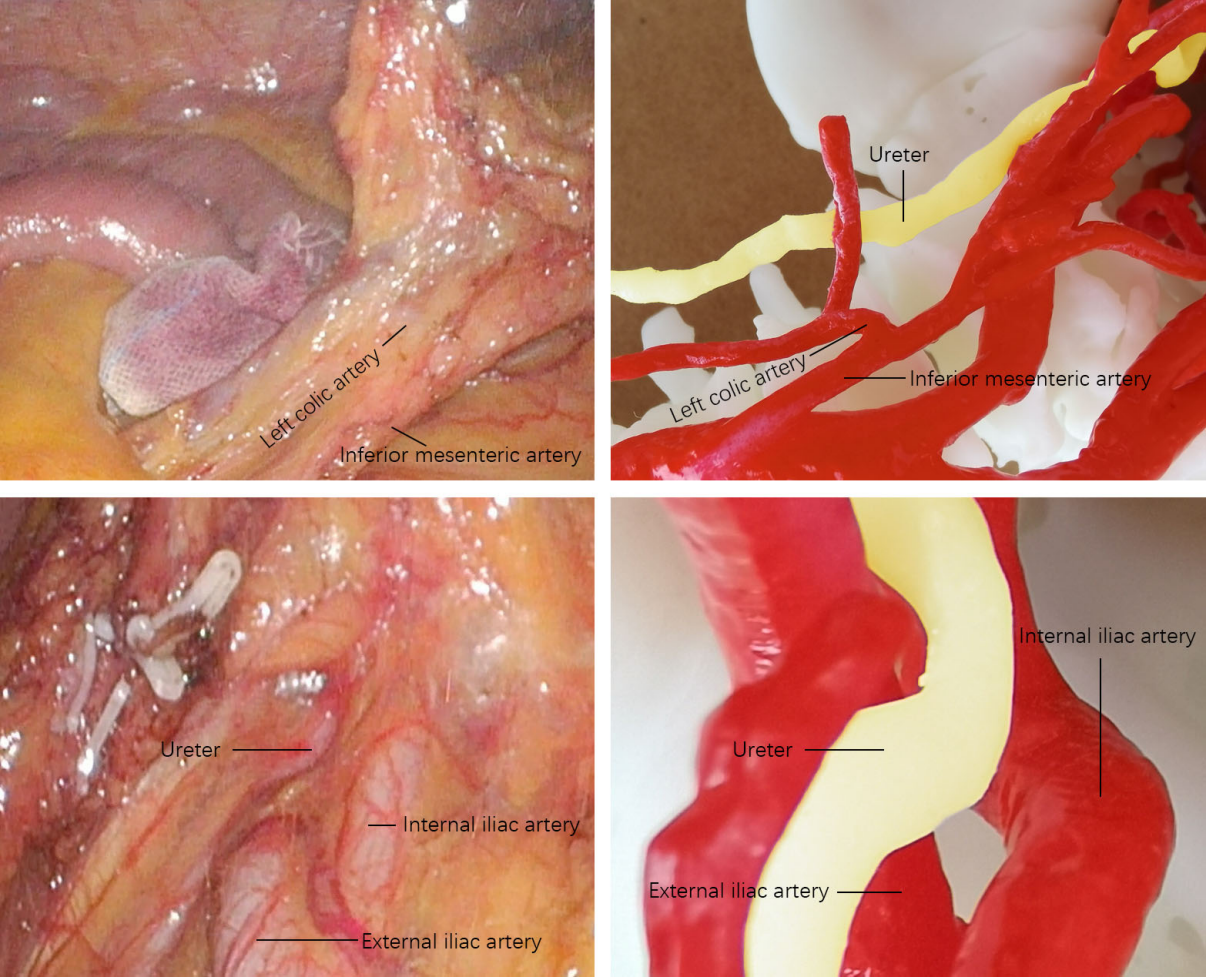
Comparison between the laparoscopic image and the pelvic model.

After acquiring CT images of arteries and veins, the 3D printing group reconstructed them into 1mm thin layer images, saved them in DICOM format and imported them into 3D modeling software for 3D virtual model and smooth the surface of the 3D virtual model. After making sure that each structure had no deformation and deviation, the resin white material was 3D printed by high-precision SLA photocuring process. After forming, curing, surface polishing and coloring are carried out. The pelvic model is shown in **Figure 1**. Each model clearly shows complex spatial relationships that, just as in a real pelvis, allow surgeons to understand these structures for preoperative evaluation and surgical planning. **Figure 2** shows a comparison of the laparoscopic images collected during laparoscopic radical resection of rectal cancer with the printed 3D pelvic model.

### Observation index

According to references (12–15), four factors were selected comprehensively to analyze the general data of the two groups, including gender, age, surgical method and clinical stage. Eight factors were selected for perioperative comparison, including operation time, intraoperative blood loss, total number of lymph nodes dissected, intraoperative time to locate inferior mesenteric artery, intraoperative time to locate left colic artery, time to first postoperative exhaust, length of hospital stay, and complications (Clavien-Dindo classification).

## Results

### Patient characteristics

There were no significant differences between the two groups in terms of gender, age, surgical methods, TNM staging (P>0.05), have comparability in **Table 1**.

**Table 1:** Patient characteristics.

Characteristic	Observation group (n=25)	Control group (n=25)	t/χ^2^	p
Sex[n(%)]				
Male	15(60)	13(52)	0.325	0.569
Female	10(40)	12(48)		
Age (years, $\bar{x}$ ± s)	59.4 ± 9.5	56.6 ± 8.7	1.070	0.290
Surgical method [n(%)]			0.149	0.700
Dixon	22(88)	20(80)		
Miles	3(12)	5(20)		
TNM staging [n(%)]			0.085	0.959
StageI	1(4)	1(4)		
StageII	11(44)	10(40)		
StageIII	13(52)	14(56)		

### Perioperative condition

Operation time, intraoperative blood loss, intraoperative time to locate inferior mesenteric artery, intraoperative time to locate left colic artery, first postoperative exhaust time and length of hospital stay in the observation group were all lower than those in the control group (P < 0.05); There were no significant differences in the total number of lymph nodes and complications between the two groups (P > 0.05) in **Table 2**.

**Table 2:** Perioperative conditions.

Characteristic	Observation group(n=25)	Control group(n=25)	t/χ^2^	p
Operation time(min, $\bar{x}$ ± s)	118.4 ± 28.2	142.7 ± 25.6	3.184	0.003
Intraoperative blood loss(ml, $\bar{x}$ ± s)	82.5 ± 19.3	126.2 ± 27.4	6.510	<0.001
Total lymph node dissection(n, $\bar{x}$ ± s)	17.2 ± 3.8	16.1 ± 2.9	1.189	0.240
Intraoperative location time of inferior mesenteric artery(min, $\bar{x}$ ± s)	7.2 ± 1.3	8.9 ± 2.1	3.338	0.002
Intraoperative time to locate the left colic artery(min, $\bar{x}$ ± s)	18.5 ± 3.8	24.7 ± 4.5	5.216	<0.001
First postoperative exhaust time(d, $\bar{x}$ ± s)	2.1 ± 1.2	2.9 ± 1.4	2.044	0.046
Stay in the hospital(d, $\bar{x}$ ± s)	9.5 ± 2.2	10.9 ± 2.5	2.033	0.048
Complications (Clavien-Dindo classification) [n(%)]				
No complications	10(40)	10(40)	0.706	0.872
GradeI	11(44)	9(36)		
GradeII	3(12)	5(20)		
GradeIII	1(4)	1(4)		

## Discussion

Surgery is one of the main methods to treat rectal cancer. Laparoscopic rectal cancer surgery has been widely used clinically with advantages such as small trauma and quick recovery. However, due to the complex pelvic anatomy, narrow space, including important digestive, urinary and gynecological organs and rich blood vessels and nerves, rectal cancer surgery still faces great challenges. Magnetic resonance imaging has become the gold standard for the evaluation of rectal cancer (16), but it can only obtain a single two-dimensional image data and is easily affected by the subjective experience of physicians. With 3D printing, a complex three-dimensional pelvic structure can be obtained from a flat two-dimensional image to accurately assess the relationship between the inferior mesenteric artery and its branches, the rectal tumor and the surrounding tissue.

In this study, the observation group had a short operation time and less intraoperative blood loss, and the difference was statistically significant (P<0.05). 3D printing pelvic model can help surgeons to establish three-dimensional and intuitive pelvic structure, more accurate positioning, clearer anatomy, shorten the time of free target blood vessels, reduce the risk of mis ligation of blood vessels, reduce intraoperative bleeding, and shorten the operation time. Mari FS (17) also believed that 3D printing technology could establish vascular models and better evaluate blood vessels, thus reducing intraoperative bleeding, which was consistent with the results of this study.

The results of this study show that 3D printing can help surgeons locate the inferior mesenteric artery and left colic artery more quickly (P< 0.05), to provide clinical decision support for surgeons, reduce intraoperative exploration time, reduce the rate of accidental injury, accelerate postoperative gastrointestinal function recovery, shorten the length of hospital stay (P< 0.05). Luzon JA (18) believed that 3D printing model has a wide application prospect as an auxiliary means of preoperative planning and a navigation tool in perioperative period. However, due to the unique physiological and volume characteristics of veins in terms of dilation and volume change, veins have the weakest correlation in perioperative period measurement.

In this study, there were no significant differences in the total number of lymph nodes dissected and the complications (Clavien-Dindo classification) between the two groups (P> 0.05). Current studies have shown that the total number of lymph node dissection is correlated with the prognosis of patients (19), indicating that there is no significant difference between the two groups in the radical treatment of tumors, and the observation group does not increase the incidence of postoperative complications, further confirming its safety. However, the study of Hojo D (20) showed that during lateral pelvic lymph node dissection, more lateral lymph nodes could be obtained through 3D printing of the pelvic model to improve the surgical effect.

In addition, in a randomized controlled trial (21), both objective evaluation and subjective questionnaire showed that 3D printing pelvic model could achieve better teaching effects than textbooks. Although 3D printing technology has many advantages in the application of rectal cancer surgery, there are still many shortcomings: (1) the manufacturing material is inelastic and cannot be pulled or valgus as in the operation; (2) Creating high-quality models requires verifying each structure on all levels of CT images, which requires a lot of labor and time; (3) It is difficult to reconstruct the neural structure; (4) More material and human resources are needed preoperatively.

## Conclusion

The application of 3D printing pelvic model in laparoscopic radical resection of rectal cancer is conducive to understanding pelvic structure and mesenteric vascular anatomy, reducing intraoperative bleeding and shortening operation time, which has a good application prospect, but it still needs to be further confirmed and evaluated by large-scale clinical studies.

## Data availability statement

The original contributions presented in the study are included in the article/supplementary material. Further inquiries can be directed to the corresponding authors.

## Ethics statement

The studies involving human participants were reviewed and approved by Medical Ethics Committee of the Second People’s Hospital of Lianyungang. The patients/participants provided their written informed consent to participate in this study. Written informed consent was obtained from the individual(s) for the publication of any potentially identifiable images or data included in this article.

## Author contributions

All authors listed have made a substantial, direct, and intellectual contribution to the work and approved it for publication.
